# The INGUIDE International Guideline Training and Certification Programme

**DOI:** 10.1002/gin2.12008

**Published:** 2024-03-11

**Authors:** Holger J. Schünemann, Robby Nieuwlaat

**Affiliations:** ^1^ Clinical Epidemiology and Research Center (CERC), Department of Biomedical Sciences Humanitas University and Humanitas Research Hospital Pieve Emanuele Italy; ^2^ Department of Health Research Methods, Evidence and Impact McMaster University Hamilton Ontario Canada; ^3^ Department of Medicine McMaster University Hamilton Ontario Canada

**Keywords:** certification, credentialing, guidelines, recommendation, taining

## Abstract

Health guidelines impact clinical, public health and policy practice, but there is no regulation for their development, often leading to variability in quality and trustworthiness. The International Guideline Training and Certification Programme (INGUIDE), established by faculty at McMaster University in partnership and under the auspice of the Guidelines International Network (GIN), addresses this shortcoming by offering structured, evidence‐based training and certification for those involved in guidelines (inguide.org). This commentary describes INGUIDE's background, purpose, structure and significance after approximately 3 years of operation. INGUIDE's mission is to enhance the quality of health guidelines globally. It provides comprehensive training to ensure the systematic development of guidelines based on the best evidence and adherence to international quality standards, as reflected in its ISO:9001 certification. The programme also emphasizes capacity building, filling educational gaps, and ensuring the global inclusivity of its courses. INGUIDE's certification covers the entire lifecycle of guideline development and includes several levels of certification, ranging from panel member to methodologist, lead methodologist, developer, chair and instructor certification. The programme already has had a global impact, training over 1500 learners since its launch. INGUIDE is led by a steering committee with input from an international advisory board and operational staff, supported by certified instructors. The programme's vision for the future includes expanding accessibility and creating additional training modules, with a commitment to continuous improvement and adaptation to diverse healthcare contexts, in particular low‐ and middle‐income countries and settings.

## Highlights

- The International Guideline Training and Certification Program (INGUIDE) is a comprehensive, evidence‐based, and up‐to‐date training program for certified guideline recommendation and development.
- INGUIDE emphasizes capacity building, filling educational gaps, and ensuring the global inclusivity of its courses.
- INGUIDE's covers the entire lifecycle of guideline development and includes several levels of certification, ranging from panel member to methodologist, lead methodologist, developer, chair, and instructor certification. The program already has had a global impact, training over 1,500 learners since its launch.
- The program's vision for the future includes expanding accessibility and creating additional training modules, with a commitment to continuous improvement and adaptation to diverse healthcare contexts, in particular low‐ and middle‐income countries and settings.

## 1 INTRODUCTION

Health guidelines produce arguably the most influential scientific publications, often cited thousands or tens of thousands of times.^1^, ^2^, ^3^ Furthermore, guidelines are some of the most wanted products of health professionals who organize in professional societies and are the source of widely used products such as UpToDate and DynaMed, thereby having an important effect on practice. However, there is ongoing concern about guideline quality and health guideline development is not regulated universally.^4^, ^5^, ^6^ Without regulation, anyone can develop a health guideline. Indeed, most people involved in guideline development, for example, through membership on a guideline development group or guideline panel, are not specifically trained to create guidelines. Yet, health guidelines should meet quality standards and their development requires a combination of clinical, public health or other content skills, research methodology training, and social and management skills.

Authorization requirements to conduct activity in the health field exist for nearly any health profession through licensing or certification, but health guideline development does not require specific certification. This creates a conundrum. Usually, highly regulated professions create guidelines, while the skills for creating them, that is, guideline science and practical aspects, are not taught in the vast majority of health professional or university programmes. The conundrum is understandable because of the highly packed curricula and the science about health recommendations in guidelines is very specialized. However, the lack of formalized training and certification is concerning, because individual health professionals may treat hundreds or perhaps thousands of people, but guideline recommendations affect most health professions and millions of people. For instance, global World Health Organization (WHO) infectious disease guidelines on tuberculosis or malaria or guidelines for cardiac or respiratory diseases developed by major professional societies or governmental organizations affect millions at a time.

Training about guideline development was typically restricted to single introductory lectures or minimal preparation. As faculty members at McMasters University working in guideline sciences, we began to think that this was a problem. We teamed up to create ‐ with and under the auspice of the Guideline International Network (GIN)—the International Guideline Training and Certification Programme (INGUIDE) (INGUIDE.org). This commentary describes INGUIDE's background, purpose, structure, and significance after approximately 3 years of operation. Additional articles will describe the methods to create the programme, how it was piloted, its detailed training and certification methods, the learners' demographics including country of origin, performance, feedback and evaluation, and other aspects of the programme.

## 2 RATIONALE FOR INGUIDE

Numerous organizations have provided standards for guideline development.^7^, ^8^, ^9^, ^10^, ^11^ While tools to plan, report and critically appraise guidelines according to these standards exist, a globally accepted certified training for guidelines as a health profession did not exist before INGUIDE.^12^, ^13^ We obtained agreement on the benefit of professional training with the GIN Board of Trustees (BoT) in 2016, and during subsequent BoT meetings our proposals were discussed and finally accepted by the GIN BoT. Following this decision, we created INGUIDE to provide standardization where possible, quality improvement and global capacity building in guideline development and implementation. Because of the available scientific and financial backing from investigator‐initiated research funding, we initiated this programme at the McMaster GRADE Center in collaboration with GIN that provided a minority investment (see below for organization and leadership of the programme). We believed that GIN was the ideal partner and umbrella organization to host such a programme because of its vision (Trustworthy and accessible guidance for better health) and mission ‘to lead, strengthen and support collaboration and work within the guideline development, adaptation, and implementation community’.

## 3 OVERVIEW, MISSION AND GOALS OF INGUIDE

INGUIDE's mission is to improve the development of health guidelines and recommendations worldwide. This is achieved through providing comprehensive, evidence‐based training to members of guideline development groups and organizations. The programme aims to elevate the standards of healthcare guideline development by offering structured, comprehensive, evidence‐based, up‐to‐date guideline development training and certification, which ensures that guidelines are developed systematically, effectively, in adherence to the highest international quality standards, and based on the best available evidence. Key elements of that mission include:

1. Improving Guideline Development Quality: There is a recognized need for a structured and standardized approach to developing health guidelines. By providing comprehensive training, INGUIDE aims to enhance the quality of guideline development, ensuring they are evidence‐based and effectively implemented.
2. Building Global Capacity: INGUIDE was established to build capacity in guideline development and implementation globally for individuals and organizations in the field of guideline development through systematic training and certification. By training individuals from various regions and backgrounds, the programme aims to elevate the standards of health guidelines worldwide.
3. Filling Educational Gaps: Before INGUIDE, there was a shortage of systematic educational programmes specifically focused on the skills and knowledge required for developing high‐quality health guidelines. INGUIDE fills this gap by offering structured, tiered certification courses.
4. Global Reach and Inclusivity: Making the programme accessible to a diverse global audience, including adapting course materials into multiple languages, and considering various international health contexts.
5. Standardization and Certification: The programme seeks to standardize the process of guideline development as much as possible and necessary and offer certification to individuals who complete the training. This standardization is important for ensuring consistency and quality in guidelines developed across different regions and healthcare systems. As a charity, GIN is an appropriate organization to oversee and conduct such certification.
6. Quality and Consistency: Ensuring that health guidelines are developed by trained professionals meet international standards of quality and consistency, as evidenced by INGUIDE's ISO:9001 certification.
7. Collaboration and Networking: INGUIDE facilitates collaboration among guideline developers, methodologists, and other interested and affected parties globally (see below for our matching service). Facilitating collaboration and sharing of best practices among healthcare professionals and organizations globally to foster a community of practice in guideline development.
8. Continuous Improvement: Committing to ongoing improvement and expansion of the programme to cover all aspects of guideline development and to stay current with the latest in evidence‐based guidelines and healthcare practices.
9. Adherence to Quality Standards: ISO:9001 is an internationally recognized standard that ensures products and services meet the needs of users and clients through an effective quality management system. It is founded on 7 quality principles (customer focus, leadership, engagement of people, process approach, improvement, evidence‐based decision making and relationship management) that INGUIDE aspires to and must adhere to with this certification.^14^ Thus, the attainment of ISO:9001 certification by INGUIDE reflects its commitment to maintaining high international quality standards in healthcare guideline development, instilling confidence in the programme's offerings.
10. Adaptation to Local Contexts: Recognizing the importance of adapting guidelines to different cultural, economic, and healthcare settings, INGUIDE includes training on how to adapt existing guidelines (through the Adolopment module) effectively and efficiently.

Thus, INGUIDE was created to elevate the quality, effectiveness, and global reach of health guideline development, providing comprehensive resources, training, and certification to those involved in the guidelines.

## 4 INGUIDE's STRUCTURE AND CERTIFICATION LEVELS

The programme covers the full lifecycle of guideline development, including planning, implementation, evaluation and updating of practice guidelines. Courses are structured according to the GIN‐McMaster Guideline Development Checklist tool and can be tailored to suit the needs of various organizations and trainees.^15^ It provides methodological skills, such as applying or understanding systematic reviews and the Grading of Recommendations Assessment, Development and Evaluation (GRADE) approach in the context of guidelines, and focuses on practical aspects, such as using the guideline participant tool and chair's checklist.^16^, ^17^, ^18^ The evidence‐to‐decision approach presents a major component of methods‐focused training.^7^, ^19^, ^20^

The organization of the programme—delivered in online, hybrid and in‐person sessions (see INGUIDE.org for details)—can be summarized as follows across, currently, four certification levels:

- Guideline Group or Panel Member Certification: Trains any individual who participates in a guideline development group or panel, including lay people. This level serves as a prerequisite for higher‐level certifications.
- Guideline Methodologist Certification: Designed for those acting as expert methodologists within a guideline development group. Certification requires relevant practical experience.
- Lead Methodologist, Guideline Developer and Chair Certification: Prepares participants to take on leadership roles such as chair or co‐chair in a guideline development group. Certification requires relevant practical experience.
- Guideline Development Instructor Certification: Focuses on training individuals responsible for educating prospective guideline development group members in any role, including methodologies and chairing skills. Certification requires relevant practical experience, and certified instructors will use quality monitored INGUIDE materials to instruct INGUIDE courses.

**Figure 1:**
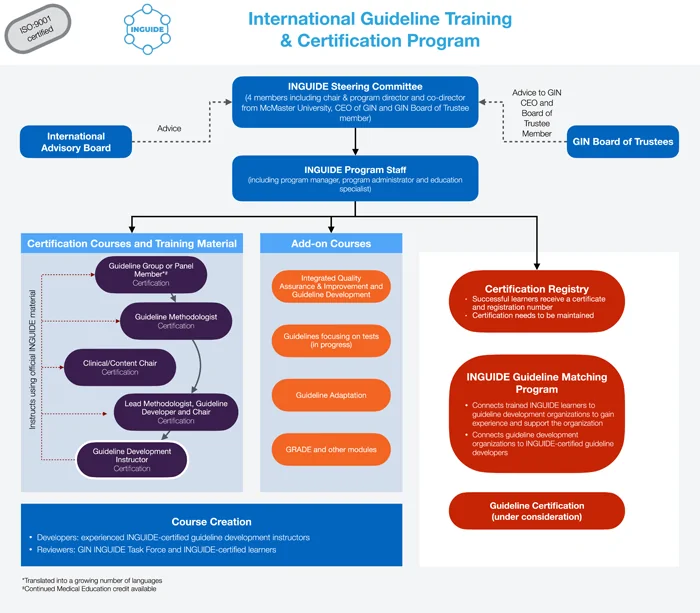
The INGUIDE international Guideline Training and Certification programme is guided by a four‐member steering committee (https://inguide.org/). The two Guidelines International Network (GIN) members on the steering committee receive advice from the GIN Board of Trustees. The steering committee as a whole also receives advice from the INGUIDE international advisory board. The INGUIDE programme staff administers the programme under direction of the steering committee. The programme includes rigorously developed and piloted core and add‐on training courses and material. New courses are developed that include various specific topics and cover GRADE. Material is developed under the supervision of the steering group or its members, by core content providers and instructors and piloted. Material is then also reviewed by the GIN INGUIDE task force. Fully certified instructors can teach INGUIDE courses using official INGUIDE material. The programme maintains a registry of certified trainees and instructors and provides a guideline‐matching service to connect guideline developers and certified or aspiring methodologists (https://inguide.org/panel-matching/). Organizations also provide peer reviews for methodologists to testify regarding their practical experiences. Certification of created guidelines is under consideration, too. INGUIDE has been ISO:9001 certified to ensure its quality.

The INGUIDE team tracks learners' progress and keeps certified individuals in a database. We previously labelled the courses as levels 1 through 4, but while advancement to the next level requires certification in the prior, we removed the numbering for at least two reasons. First, most learners, intentionally, will only complete the guideline group or member certification course because that is where their involvement with guidelines stays. Second, INGUIDE also has special focus modules. As of late 2023, these include a quality assurance and a GRADE adolopment module.^21^, ^22^ A specific certification course for clinical and content chairs that will focus on the group leadership part of the lead methodologist and chair certification is nearly completed and future modules will include more specialized training, including on testing and diagnosis and more specific GRADE modules. To create these modules and training opportunities as well as ISO:9001 certification requires time and effort, but we have ambitious timelines. Through an increasing number of certified instructors, our goal is to increase the global capacity in creating these quality‐assured training opportunities. The figure provides an overview of the organization, oversight, training courses, certification approach and contributors to the INGUIDE programme (Figure 1).

## 5 OTHER CORE COMPONENTS

We developed a guideline‐matching service to facilitate the alignment of training and resources with the needs of individuals and organizations (https://inguide.org/panel-matching/). This service allows both trained individuals and organizations seeking INGUIDE‐trained individuals to connect. We felt that obtaining ISO:9001 Certification demonstrates the programme's commitment to international quality standards in health guideline development, that is, the quality of our programme is monitored independently.

## 6 GLOBAL IMPACT, COLLABORATIONS AND TRANSLATIONS

Since the launch of the INGUIDE programme, over 1500 learners have been trained across its various certification levels. Specifically, the programme has seen more than 1,200 learners enroled in the ‘Guideline Group or Panel Member Certification’ since its inception in September 2020. Additionally, the ‘Guideline Methodologist Certification’ has enroled over 300 learners since its launch in May 2021, over 60 individuals have been trained for “Lead methodologist, guideline developer and chair certification” since its launch in September 2022, and 10 individuals have been trained as guideline development instructor since September 2023. A separate publication will describe the experience, feedback, and demographics of these global learners. We specifically partnered with the European Commission to develop the quality assurance and improvement module and with the WHO to support guideline adolopment through a dedicated module. Furthermore, we have a partnership with the Canadian Royal College of Physicians and Surgeons and offer continuing medical education credits. In addition, we have translated material into Chinese, French and Spanish and are conducting translation efforts in several other languages to create global capacity of certified guideline developers.

## 7 SUCCESS STORIES AND TESTIMONIALS

We already have numerous success stories and learnings. One of our early partners, the Endocrine Society trained their methodologists and guideline panel members through INGUIDE.^23^ Furthermore, because of their commitment to quality guideline development group members of major organizations have been trained, such as the European Commission, the WHO, the Public Health Agency of Canada, the Endocrine Society and the European Academy of Allergy & Clinical Immunology (EAACI) to name a few. Testimonials from trainees or organizations that have benefited from the programme are listed on our website (https://inguide.org/testimonials/). These success stories will be described in more detail in future articles. We also have learned that the developments in the guideline science will require updating of courses—something we have already done—and that language translations require careful attention to keep material consistent between different courses. We also created an international advisory board to ensure we keep the programme aligned with global developments.

## 8 ORGANIZATION AND LEADERSHIP OF THE PROGRAMME

The organizational structure of the INGUIDE programme is comprehensive and multi‐faceted, encompassing various teams and components. The structure includes:

a) Steering Committee: This is the top‐level management responsible for overall strategic direction and decision‐making and includes the two science leads (authors of this article, HJS chairs the steering committee and is programme director, RN is programme co‐director), the CEO of GIN (Elaine Harrow), and a GIN BoT member (as of 1 January 2024 Roberta James) (https://inguide.org/inguide-team/steering-committee/).
b) International Advisory Board: A group of international experts providing input to the INGUIDE steering committee and programme, ensuring it aligns with best practices and meets its objectives (https://inguide.org/inguide-team/international-advisory-board/).
c) Operational Staff: The team handles the day‐to‐day operations of the programme, including administration, logistics, finances, websites, certification monitoring and coordination of activities (https://inguide.org/inguide-team-members/).
d) Core instructors: These individuals are responsible for delivering the educational content of the programme, supported by a digital pedagogy specialist, ensuring it is comprehensive, up‐to‐date, and effective for learners. This group includes core faculty at McMaster University and other universities but is growing rapidly as the number of certified instructors rises (https://inguide.org/inguide-team-members/).

## 9 FUTURE DIRECTIONS AND ASPIRATIONS

Operation of the programme is costly and time‐consuming. Our vision for the future impact of INGUIDE on global health guidelines and recommendation development is that we are seeking independent funders to support us in offering capacity building globally at no or minimal cost to participants in low‐ and middle‐income countries and settings. In addition, accessibility is enhanced by offering courses in various languages, which requires careful coordination and resources. Furthermore, we are looking at developing additional modules to cover the breadth of guidelines development. Readers are encouraged to connect with us and suggest priorities to help us create that strategy (https://inguide.org/contact/).

## 10 CONCLUSION

In summary, INGUIDE was created to elevate the quality, effectiveness, and global reach of healthcare guideline development, providing comprehensive resources, training, and certification to guideline creators under the auspices of GIN, a Scottish registered charity.

## AUTHOR CONTRIBUTIONS

**Holger J. Schünemann**: Conceptualization; funding acquisition; writing—original draft; resources; writing—review and editing; methodology; visualization; project administration. **Robby Nieuwlaat**: Writing—review and editing; conceptualization; methodology; resources; visualization; project administration.

## CONFLICT OF INTEREST STATEMENT

This commentary was invited. Holger J. Schünemann (HJS) is member of the Editorial Board of Clinical and Public Health Guidelines, but he did not participate in the editorial process of this paper and had no influence on the editorial decisions related to it. HJS and Robby Nieuwlaat (RN) are creators of INGUIDE, members of the steering committee and programme directors of the INGUIDE programme. HJS is chair of the steering committee and co‐chair of the GRADE Working Group. HJS and RN conducted the work until now while being at McMaster University. HJS now maintains an emeritus appointment at McMaster University but his primary appointment is at Humanitas University, Milan, Italy. Coincidentally, HJS is the current chair of the Guidelines International Network (GIN) and is recused from decisions about INGUIDE at GIN board of trustees meetings. They have not received direct payments through the INGUIDE programme.

## ETHICS STATEMENT

The authors did not seek ethics board review for this invited commentary.

## Data Availability

The data that support the findings of this study are available from the corresponding author upon reasonable request.
